# The mediating role of hope in the relation between uncertainty and social support with self-management among patients with ESKD undergoing hemodialysis

**DOI:** 10.1186/s12882-024-03558-2

**Published:** 2024-04-12

**Authors:** Ali Mohammad Parviniannasab, Fatemeh Dehghani, Seyyed Ali Hosseini

**Affiliations:** 1 Department of Nursing, School of Nursing, Larestan University of Medical Sciences, Larestan, Iran; 2 Bachelor of Science in Nursing, Larestan University of Medical Sciences, Larestan, Iran; 3 School of Nursing, Larestan University of Medical Sciences, Larestan, Iran

**Keywords:** Hemodialysis patients, Self-management behavior, Social support, Uncertainty, Hope, Mediating effect

## Abstract

**Background:**

Self-management behaviours are critical for patients requiring regular hemodialysis (HD) therapy. This study aimed to test the relationship between social support, uncertainty and self-management among HD patients and to explore whether hope plays a mediating role.

**Methods:**

In a cross-sectional study, a convenience sample of 212 HD patients from two hospitals completed the Perceived Social Support Scale (PSSS), Herth Hope Index (HHI), Short form Mishel Uncertainty in Illness Scale (SF-MUIS), and hemodialysis Self-Management Instrument (HD-SMI). Data were analysed using structural equation modelling.

**Results:**

The main finding indicated that social support positively affected self-management (β = 0.50, t = 4.97, *p* < 0.001), and uncertainty negatively affected self-management (β =-0.37, t=-4.12, p = < 0.001). In mediational model analysis, the effect of social support on self-management was fully mediated [(β = 0.12; 95% BC CI (0.047, 0.228)] by hope. Also, the effect of uncertainty on self-management was fully mediated [(β=- 0.014; 95% BC CI (-0.114, -0.003)] by hope.

**Conclusions:**

“Considering factors influencing self-management in HD patients is crucial for improving quality of life. Receiving support and informational resources can not only foster hope but also reduce their uncertainty, thus aiding in enhancing clinical outcomes, quality of life, and reducing complications. “Health care providers, especially nurses were advised to accept the existence of uncertainty, help patients make optimal use of support resources, and give more importance to disambiguation to reassure them. Therefore, well-designed interventions that enhance social support and hope and reduce uncertainty may help improve self-management behaviour in HD patients.

## Background

End-stage kidney disease (ESKD) is a life-threatening and chronic condition in which the kidney function is no longer adequate to support the body’s needs [1, 2]. It is estimated that more than two million patients worldwide are being treated for ESKD [3]. However, it is essential to note that hemodialysis (HD) can only substitute for about 10% of renal function [4]. The administration of HD medication has been found to enhance survival rates and prolong life expectancy. However, patients undergoing this treatment encounter many problems encompassing both physical and mental aspects. These challenges not limited to increase blood urea nitrogen, serum potassium and phosphate levels, Dietary and fluid non-adherence, but they also suffer from poor sleep quality, sexual dysfunction, fatigue, stress, depression, uncertainty, and anxiety [5–8]. While HD is effective in preserving physical function and mitigating problems in individuals with chronic kidney disease, it is challenging to assert that HD alone is solely responsible for improving the patient’s overall state. The management of this disease necessitates the adoption of self-management behaviours by patients to enhance their well-being [9]. Thus, an effective way to reduce mortality and complications and improve quality of life is to improve self-management among patients undergoing HD [10]. Self-management, in the context of ESKD, refers to an individual’s ability to alleviate symptoms, manage treatment side effects, handle psychosocial effects, regulate emotions, and make necessary lifestyle changes to maintain a satisfactory quality of life and reduce medical expenses [11, 12]. According to social cognitive theory (SCT), self-management is a form of health behaviour influenced by individual and environmental factors [13], Uncertainty [14], social support [15], and hope have been reported as factors that influence self-management in patients with ESKD [8, 16, 17].

As an environmental factor, social support improves self-management [18]. Social support is received from a network of individuals and social groups such as family, friends, and healthcare providers (HCP) [19]. A study on ESKD patients undergoing HD found a significant relationship between social support and self-management, highlighting the importance of functional social support as an integral part of self-management intervention [20]. Research has shown that positive social support enhances self-regulation and participation in self-management behaviors [21]. Social support through peer support programs in previous studies has been evident in improving adherence to treatment management in chronic conditions [22]. Other study results have demonstrated that the availability of social support can impact overall self-management, problem-solving, and emotional management [10].

Uncertainty is another environmental factor that affects self-management. Originating from Michel’s uncertainty theory [23], it is particularly prevalent among patients undergoing HD due to the unpredictable nature of disease progression and treatment outcomes. Several quantitative studies have found a negative correlation between disease uncertainty and self-management behaviors in patients undergoing long-term hemodialysis. However, the topic of predicting disease uncertainty during hospitalization in relation to self-management behaviors has rarely been discussed [24]. Other study results also suggest that uncertainty contributes to reduced self-care behaviors [25].

Hope, which is closely related to self-management [26], is a motivational and dynamic psychological process that serves as a healing and powerful agent for facing the future with a positive attitude [27]. Previous studies have emphasized hope’s importance as a mediator and an adaptive mechanism in chronic diseases [28–30]. It is possible to postulate that hope mediates the association between social support and uncertainty concerning self-management. Hope is a cognitive construct encompassing a belief in attaining a desired outcome in the foreseeable future and a cognitive, affective state that influences individuals’ actions [31]. Snyder’s theoretical framework on hope further shows the capacity of hope to facilitate self-regulatory actions in individuals [32]. Furthermore, prior research has demonstrated a notable positive correlation between hope and social support, with social support as a predictor of hope levels [33]. However, the intricate nature of the treatment process, the length of treatment, the potential trajectory of the treatment process, and the management of adverse effects associated with HD give rise to a sense of ambiguity. In a research investigation, it has been observed that hope plays a significant role in safeguarding individuals against the detrimental impacts of chronic and persistent stress associated with disease [8]. Hope is an essential internal resource for chronic patients under acute and chronic stress because it promotes self-management behaviours and improves quality of life [34]. Therefore, the possibility that hope plays a mediating role between social support and uncertainty with self-management needs to be identified. thus, we proposed the following hypotheses:

### Hypothesis 1

Social support is positively related to self-management (Total effect1).

### Hypothesis 2

Uncertainty is negatively related to self-management (Total effect2).

### Hypothesis3

**Fig. 1:**
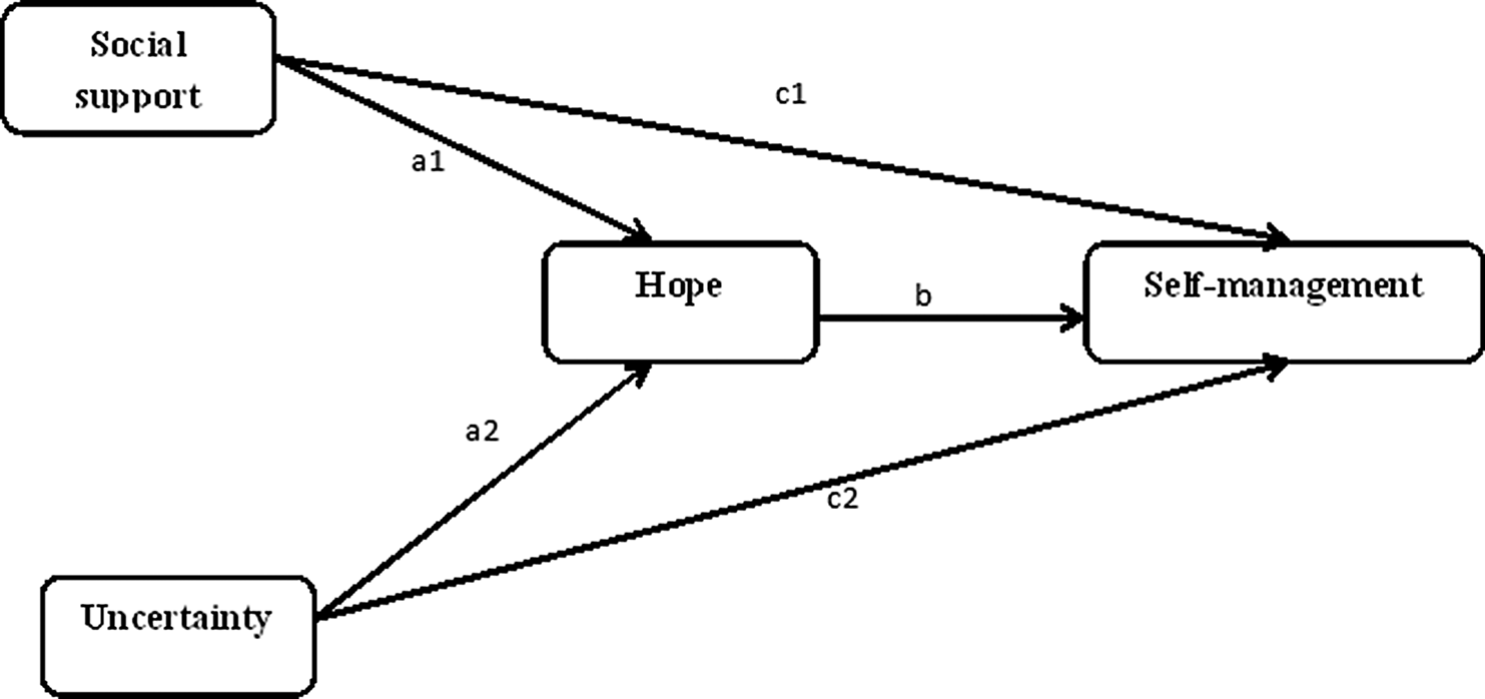
Hypothesized mediated model. *c*1: total effect1; *c*2: total effect2 (without considering role of the mediator); *a1, a2*: effects of the predictor on the mediator; *b*: effect of the mediator on the outcome; *a1b*: indirect effect 1; *a2b*: indirect effect 2

Social support (Indirect effect 1) and uncertainty (Indirect effect 2) affect self-management through hope ((Mediation Effect). The hypothetical model is shown in Fig. 1.

## Methods

### Study design, setting and participants

This cross-sectional study was carried out on 212 participants with ESKD Undergoing HD. Participants were recruited from the HD centres affiliated with Larestan and Shiraz University of Medical Sciences in Fars province in the south of Iran from April to July 2023. A convenience sampling method was used to select participants for this study. Inclusion criteria were (1) 18 years or older, (2) under maintenance HD for more than three months, (3) being able to read and write, (4) hemodialysis performed 2–3 times a week, and (5) willingness to participate in the study. Participants with acute illness or hospitalization and those reporting mental or cognitive impairment or physical limitations in self-care were excluded. According to the study conducted by Wolf et al. (2012) [35], in using structural equation modeling, the sample size is calculated based on the estimation of free parameters in the model, with a requirement of 5 to 10 observations per free parameter. Since we had 36 free parameters in the model, a minimum sample size of 180 is needed. Therefore, sampling was conducted in the designated centers, and we calculated a minimum of 200 cases based on the principle of minimum required numbers for conducting structural equation modeling (SEM) [36]. Additionally, Kline (2023) suggested considering 5 to 20 observations per parameter [37]. Hence, considering potential attrition, we increased the sample size to 226 individuals. After excluding incomplete questionnaires, we included 212 questionnaires in the final analysis.

### Data collection

From April to July 2023, a research assistant with expertise in the specific field under investigation delivered questionnaires to participants who met the defined criteria at the designated center, in a quiet room before undergoing hemodialysis. Before distributing the questionnaires, participants signed an informed consent form, emphasizing the importance of maintaining anonymity and confidentiality of their responses. Each individual completed the questionnaire in a separate room to ensure their answers were not influenced by others. During the completion of the questionnaire, the responsible data collector clarified any ambiguities in the questions. The completion of the questionnaires took between 20 and 30 min. Participants returned their completed questionnaires on the same day.

### Measurements

The initial measurements included general demographic questions covering gender (male and female), age, education level, employment status, and marital status. Additionally, questions related to hemodialysis such as the number of dialysis sessions per week, years under hemodialysis, and comorbidities were included. Furthermore, four main scales measuring self-management, social support, uncertainty, and hope were provided to participants for completion. These instruments were in English and had been previously translated for use in various studies in Iran. Therefore, we utilized instruments with sufficient reliability and validity that are widely used.

### Hemodialysis self-management instrument (HD-SMI)

Song first designed this instrument in 2009 [38], and Li et al. in 2014 [10] also used it in their research (Song, 2009; Li et al., 2014). This scale consists of 20 items divided into four subscales, including (1) problem-solving (five items), (2) emotion management (four items), (3) self-care (seven items) and (4) partnership (four elements). Items are rated based on a 4-point Likert scale ranging from 1 = never to 4 = always. Total scores range from 20 to 80. Higher scores indicate better self-management in the patient. In the original version, Cronbach’s coefficient of the overall scale is 0. 87 [38]. In Iran, the content validity (confirmed by 10 members of the faculty), face validity (by distributing the scale among 30 patients), and reliability of this tool were confirmed in the study by Hafezieh et al. in 2020 [39].The Cronbach’s α in the current study was 0.88.

### Herth hope index (HHI)

This scale consists of 12 and is distributed into three subscales: temporality and future, cheerful readiness and expectancy, and interconnectedness. Each item was scored on a 3-point Likert scale from 1 (disagree) to 3 (agree), but items 3 and 6 required reverse scoring. Total scores range from 12 to 36. higher scores indicate a higher level of hope. The internal consistency reliability was good in our sample (α = 0.83) [40]. In Iran, Pour ghaznein (2005) utilized the two mentioned methods for determining the reliability of the Herth tool after its extraction and translation. Based on this, the Cronbach’s alpha coefficient was determined to be 0.76, and the Pearson correlation coefficient was 0.84. Additionally, the content validity of the tool was confirmed through the adaptation of each question to the dimensions of the Hope Default Scale and the consensus of several psychology experts. This scale has also been used in kidney transplant patients, and its reliability has been reassessed through retesting. A Pearson correlation coefficient of 78% was obtained, confirming the reliability of the tool again [41]. The Cronbach’s α in the current study was 0.88.

### Multidimensional scale of perceived social support (MSPSS)

This scale was first developed by Zimet in 1988 [42]. **MSPSS** consists of 12 items and three subscales: family support (four items), friends support (four items), and other support (four items). Each item is rated on a five-point scale, ranging from 0 (strongly disagree) to 4 (strongly agree). The total scores range between 0 and 48. Higher scores indicate higher perceived social in the patient. As the total score increases, the level also increases. The Cronbach’s alpha coefficient for the original version was 0.85. In Iran, the content validity (confirmed by 10 members of the faculty), face validity (by distributing the scale among 30 patients), and reliability of this tool were confirmed in the study by Besharat et al. (2019) [43]. The validity and reliability of the MSPSS tool have been sufficiently demonstrated in the Iranian community. The Cronbach’s alpha coefficient was 0.84 for the scale and 0.85 or higher for each of its domains, and it remained sufficiently stable after a two-month period (0.84) [43]. The Cronbach’s α in the current study was 0.87.

### Short form mishel uncertainty in illness scale (SF-MUIS)

The SF-MUIS covers five statements from the modified 33 questions the Mishel Uncertainty in Illness Scale [44], examining the uncertainty in illness in hospitalised adults [45]. This scale consists of 5 items. Items are rated based on a 5-point Likert scale ranging from 1 = strongly disagree to 5 = strongly agree. Total scores range from 5 to 25. Higher scores indicate Higher uncertainty in the patient. The coefficient of Cronbach’s alpha in the original version was 0.7 [45]. The test-retest of the SF-MUIS was 0.98. Alizadeh et al. (2018) translated the SF-MUIS into Persian and evaluated its psychometric properties and reported the content validity ratio and content validity index of the tool to be 0.8 and 0.97.4, respectively. They also reported the Cronbach’s alpha of the SF-MUIS was 0.89 [46]. The Cronbach’s α in the current study was 0.90.

### Ethical consideration

The present study was approved by the local Ethics Committee of Larestan University of Medical Sciences, Larestan, Iran (ethical code: IR. LARUMS. REC.1401.009). Written informed consent was obtained from all the participants. All participants were informed of the aim and methods of the study; they were also assured about the anonymity and confidentiality of their data.

### Data analysis

participants’ demographic characteristics were described using frequency, percent, mean ± standard deviation, kurtosis, and skewness. Pearson correlation using SPSS 24.0 software analysis was used to examine the relationship between study variables. The structural equation model (SEM) using AMOS 24.0 with maximum likelihood estimations was utilised to test the structural equation model. The following fit indices have been used to assess the quality of the model: (χ2/df) < 5 [47], root mean square error of approximation (RMSEA) < 0.080, standardised root mean square (SRMR) < 0.080, Goodness of-fit index (GFI) > 0.90, comparative fit index (CFI) > 0.90, and Tucker–Lewis Index (TLI) > 0.90 [48].

## Results

### Descriptive statistics

Of the 212 participants, 127 were men (59.9%) and 85 were women (40.1%). The average age of the participants was 49.82 ± 16.024 years. 162 (76.4.5%) were married, and most (58.5%) were unemployed. Most participants, 123 (58%) had high school or higher education. The weekly HD frequency was 3 in 140 (66%) participants. Moreover, 38.2% of the participants received HD therapy for over 60 months, and 51.4% had one or two comorbidities in addition to kidney disease (Table 1).

**Table 1:** Demographic characteristics of the participants (*N* = 212)

Characteristic	Categories	N (%)
Gender	Male	127 (59.9)
	Female	85 (40.1)
Age	Range (year) = 18 ~ 86. Mean ± SD = 49.82 ± 16.024	
Marital status	Married	162 (76.4)
	Others	50 (23.6)
Educational level	< High school diploma	55 (26)
	≥High school diploma	123 (58)
	Illiterate	34 (16)
Hemodialysis frequency/week	1–2 times	73 (34)
	3 times	140 (66)
Duration of dialysis (month)	< 12	47 (22.2)
	12–60	84 (39.6)
	> 60	81 (38.2)
Number of comorbidities	0	82 (38.2)
	1–2	109 (51.4)
	3–5	22 (10.4)
Employment status	Employed	25 (11.8)
	Unemployed	124 (58.5)
	retriving	63 (29.7)

### Bivariate correlation analysis

Table 2 shows the study variables’ means, standard deviations, and correlations. Social support is positively and significantly related to self-management (*r* = 0.39, *p* < 0.01) and hope (*r* = 0.38, *p* < 0.01). uncertainty is negatively and significantly related to self-management (*r* = -0.35, *p* < 0.01) and hope (*r* = -0.19, *p* < 0.01). finally, hope (*r* = 0.38, *p* < 0.01) is positively and significantly related to self-management.

**Table 2:** Correlations, means and standards deviations of study variables

	M	SD	1	2	3	4
Social Support	34.94	9.55	1			
Uncertainty	11.90	4.98	0.012^ns^	1		
Hope	26.8	4.82	0.38^**^	-0.19**	1	
Self-management	61.53	9.22	0.39^**^	-0.35^*8*^	0.38^**^	1

### Path analysis of effects of social support and uncertainty on self-management

The structural equation model (SEM) was used to measure the variables’ relationship. First, we tested the normality using skewness (-3, + 3) and kurtosis (-3, + 3), which was established; based on the data, the skewness values for social support, hope, uncertainty, and self-management were found to be -0.80, -1.42, 0.69, and − 1.48, respectively. The variables exhibited absolute kurtosis values of 0.86, 1.47, -0.34, and 2.38, respectively. Therefore, the obtained results are consistent with the assumption of normal distributions [49]. The last phase involved the implementation of confirmatory factor analysis to assess the measurement model’s validity by analysing the associations between the observed variables and the latent constructs. Typically, a factor loading of 0.40 or higher is considered satisfactory [40]. As a result, two items from the hope measurement model and two from the self-management measurement model were excluded from the final analysis because their factor loadings were less than 0.4. After removing items with low factor loadings (less than 0.40), fit indices were calculated for the structural model. The adequacy of the suggested model fit was assessed using several indices: χ2/df = 1.76, RMSEA = 0.060, GFI = 0.913, TLI = 0.951, CFI = 0.960, and SRMR = 0.059, indicating that the fit indices were satisfactory. In order to enhance the model fit, a pair of covariance parameters in the uncertainty was incorporated for modification indices greater than 5. The resulting model exhibited improved indices (χ2/df = 1.68, RMSEA = 0.057, GFI = 0.919, TLI = 0.959, CFI = 0.965, SRMR = 0.060). The results indicate that social support significantly impacted self-management (β = 0.50, t = 4.97, *p* < 0.001), supporting Hypothesis H1. Uncertainty had a detrimental impact on self-management, as indicated by the significant negative beta coefficient (β = -0.37, t = -4.12, *p* < 0.001). This finding provides support for Hypothesis H2, as presented in Table 3.

**Table 3:** Total effects of social support and uncertainty on the self-management (*n* = 212)

Effects	Paths	β standardized	SE	t	p
Total Effect1: c1	H1: SS SM	0.509	0.024	4.97	0.001^***^
Total Effect2: c2	H2: UN ◊SM	-0.370	0.067	-4.12	0.001^***^

***p< 0.001, **p<0.01, ns: not significant; SS, social support; SM, self-management; UN, uncertainly

### Mediational model analysis

**Fig. 2:**
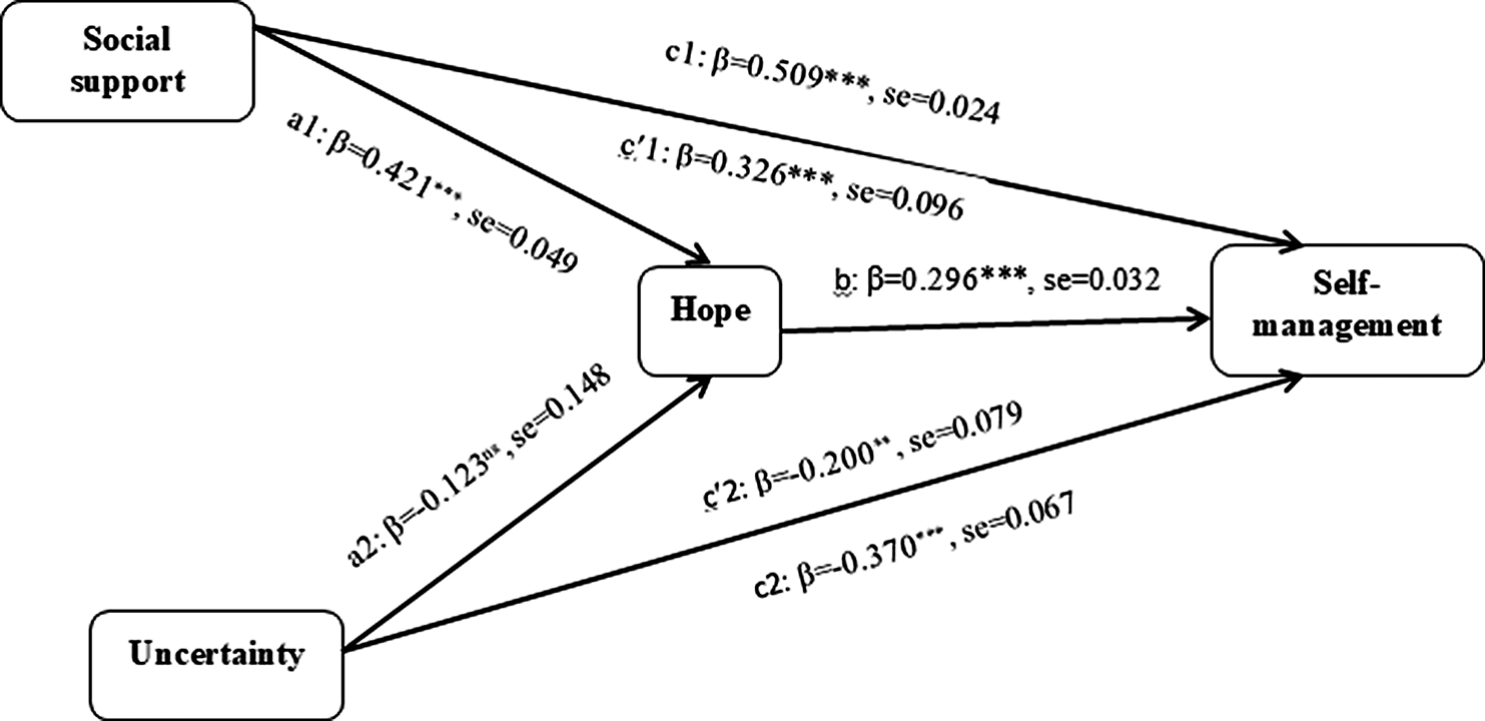
**c1 and c2**, total effect of predictors (social support and uncertainty) on outcome(self-management) without considering role of the mediator; ***c*‘1 and*c*’2**, direct effect of predictors on outcome while considering role of the mediator; ***a1 and a2***, effect of the predictors on the mediator; ***b***, effect of the mediator on the outcome; ***a1b*and a2b**, indirect effects of social support and uncertainty on self-management through hope, respectively; ***p< 0.001, **p<0.01, ns, not significant;

For the final hypothesis, we used the bias-corrected bootstrap 95% confidence interval based on 5,000 bootstrapping to test the mediating effect [50]. social support had an indirect effect on self-management through hope [(β = 0.12; 95% BC CI (0.047, 0.228)]. Additionally, social support had direct effects on self-management through hope [(β = 0.32; 95% BC CI (0.132, 0.505)], indicating the full mediation effects of hope (H3). The uncertainty had an indirect effect on the self-management through hope [(β=- 0.014; 95% BC CI (-0.114, -0.003)]. Additionally, uncertainty had direct effects on self-management through hope [(β=- 0.200; 95% BC CI (-0.365, -0.056)], indicating the full mediation effects of hope (H3) (see Table 4). Figure 2 shows the final model with standardized path coefficients.

**Table 4:** Results of mediation testing: bootstrap analysis

Mediation Hypothesis			Effect	BootSE	p	Boot LLCI 95%	Boot ULCI 95%	Result
H3	SS→hope→SM	Indirect effect1	0.121	0.045	0.000^***^	0.047	0.228	Full Mediation
		Direct effect1	0.326	0.096	0.000^***^	0.132	0.505	
	Uncertainly →hope→SM	Indirect effect2	-0.014	0.027	0.028*	-0.114	-0.003	Full Mediation
		Direct effect2	-0.200	0.079	0.008**	-0.365	-0.056	

***p< 0.001, **p<0.01, *p<0.05, ns, not significant; SS, social support; SM, self-management; LLCI, lower level of confidence interval; ULCI, upper level of confidence interval

## Discussion

The results showed that hope completely mediates the relationship between social support and uncertainty with self-management. In confirmation of the first hypothesis, social support positively and significantly predicts self-management. This finding is similar to previous studies [14, 18, 51], which showed that support from family, friends, and peers positively affected self-care behaviours. These results suggest that coping strategies, such as creating a support system with family and health professionals beyond simply improving disease-related behaviours, are necessary to increase self-management behaviours [52]. Also, previous findings showed that patients with better support are more likely to have more positive mental states and solve problems with better use of available resources [21, 53]. Better social support helps HD patients adhere to treatment regimens, such as diet and fluid management, which are considered the most important self-management behaviours in HD patients [9, 54]. It seems, HD patients’ self-management behaviours can be influenced by members of social networks, especially family and caregivers [55].

In line with the second hypothesis, the results showed that uncertainty negatively and significantly predicts self-management. These results are consistent with the results of a previous study [14]. it can be claimed that HD patients due to the complexity of their disease and treatment process and experience problems such as depression and anxiety because they cannot predict the progress of the disease or complications that may occur [56], in turn, has a negative effect on self-care and health-promoting behaviours of HD patients [57]. Thus, the unpredictable progress of the disease and treatment process can lead to weakness in improving self-management behaviours [7]. Therefore, to reduce the uncertainty of HD patients and ultimately increase their level of self-management, health professionals can provide patients with detailed explanations about the disease and treatment [58].

In line with the third hypothesis, hope has a positive and significant relationship with self-management. The results of a study indicate that patients’ level of hope is positively associated with self-management, suggesting that patients with higher levels of hope are likely to adhere to treatment and effectively demonstrate self-initiated behaviors, consistent with previous findings [59]. Hope, as a positive internal dynamic force, is an important strategy for patients to cope with illness. Existing studies suggest that a good level of hope can mobilize positive emotions and self-efficacy in individuals [60]. Patients with higher levels of hope can better resist the harms caused by negative emotions and have expectations and perseverance for treatment and rehabilitation. Therefore, they have a strong belief in successfully executing and completing a specific behavioral goal, which makes patients more inclined to actively learn and collaborate with others to take positive actions to cope with the disease and improve their physical condition [34, 61].

In addition, it completely mediates the effect of social support and uncertainty on self-management. The study of Zhang in 2023 in liver transplant patients showed that receiving social support and a robust social network system increases hope and provides a better outlook for patients. Consequently, it encourages people to take care of themselves, which can increase their level of self-management [62]. In many other studies, hope plays a mediating role between psychological variables [28, 29]. Social support can help the patient choose a positive coping strategy. This situation increases hope by reducing the distress caused by symptoms. It was also shown that if a person has the support of peers, he can deal with difficult situations caused by the disease, and as a result, he can enjoy an advanced emotional state, which in turn can increase the level of hope of the person [34]. Stating that hope can be effective in the psychological characteristics of patients, hope is a powerful coping mechanism in patients with chronic diseases, and hopeful people can bear the damage caused by the disease more efficiently and have better self-management behaviours [63].

Finally, uncertainty through hope has a negative effect on self-management behaviours. Uncertainty causes a person to have poor management in not very specific situations [64]. In a study conducted on cancer patients, hope is a mediator against adverse and stressful events in the cancer experience. Therefore, the literature shows that hope has a protective effect on chronic disease by reducing distress [30]. A study conducted on heart failure patients reported that disease uncertainty as perceived by the patient has a negative and indirect effect on quality of life through perceived stress and acceptance/rejection [57]. It seems reducing uncertainty in PD patients not only requires providing them with information but also improving their mental understanding of health and assisting them in maintaining hope when they feel their health is deteriorating. Since PD nurses often interact with patients, they are in the best position to help reduce patient uncertainty. Furthermore, nurses should be aware of patients’ need to maintain hope and a positive outlook on their health status, even if patients do not directly discuss these issues [65]. Based on the aforementioned findings, healthcare providers should emphasize the importance of positive self-efficacy and hopefulness for behavior change in patients. Nursing staff can encourage patients to confront their illness through health education or psychological intervention, correct their misconceptions such as “illness equals incapacity,” and help patients understand their personal empowerment in delaying the progression of the disease. This way, fostering patients’ self-management flexibility and enthusiasm [25].

### Implication for practice

For ESRD patients undergoing HD who require long-term or even lifelong regular treatment, self-management is critical to their health outcomes. This study confirms for the first time that social support and uncertainty directly affect self-management in this population and has identified the mediating role of hope in the relationship between these variables. HCPs should consider social support and hope when designing interventions to enhance self-management in HD patients. Also, explaining the lifestyle, therapeutic and nutritional regimen can reduce ambiguities and provide the basis for better disease control.

### Limitations

The present study is subject to many limitations. Initially, the researchers employed convenience sampling, which may restrict the generalization of the research findings. Therefore, it is imperative to replicate the present study using bigger sample sizes randomly selected from various ethnic subpopulations, socio-economic groups, and broader geographical areas. Furthermore, it is essential to note that the present study employed a cross-sectional design. Hence, the determination of causal connections is rendered inconclusive. Hence, it is imperative to conduct additional longitudinal studies to ascertain causation and examine the association between these variables. Furthermore, it is essential to note that while the study was voluntary and participants were guaranteed confidentiality, the potential for response bias cannot be overlooked. This is particularly relevant considering the sensitive nature of the participants’ situations. This phenomenon is a frequently encountered constraint in self-report questionnaires. Hence, it is recommended that future research endeavours incorporate performance-based instruments in addition to self-report scales to assess the variables under investigation. The omission of considerations about socio-cognitive qualities, coping methods, and other psychological disorders may have an impact on the association between social support and uncertainty concerning self-management. Hence, future research endeavours should delve into these situations.

## Conclusions

The present study sheds light on the association between social support and uncertainty about self-management, with the mediating role of hope. These findings are essential for developing intervention techniques to enhance self-management behaviours. Nurses were instructed to acknowledge the presence of uncertainty and assist patients in maximising their use of support systems while emphasising the significance of disambiguation as a means of reassurance. Providing emotional support and disseminating better and more consistent information, particularly about the individual’s illness status, symptoms, treatment progress, and prospects in terms of disease control, with opportunities for open discussion of their concerns, can be of significant use. Consequently, treatments that are carefully constructed to augment social support and hope while simultaneously mitigating uncertainty have the potential to boost self-management behaviour in HD patients.

## Data Availability

The datasets used and/or analysed during the current study are available from the corresponding author on reasonable request.

## Abbreviations

ESRD: End-stage renal disease
HD: Hemodialysis
HCP: healthcare providers
HHI: Herth hope index
PSSS: Perceived social support scale
SEM: Structural equation model
χ2/df: chi-square / degrees of freedom
RMSEA: Root mean square error of approximation
SRMR: Standardised root mean square
GFI: Goodness of-fit index
CFI: Comparative fit index
TLI: Tucker–lewis index
